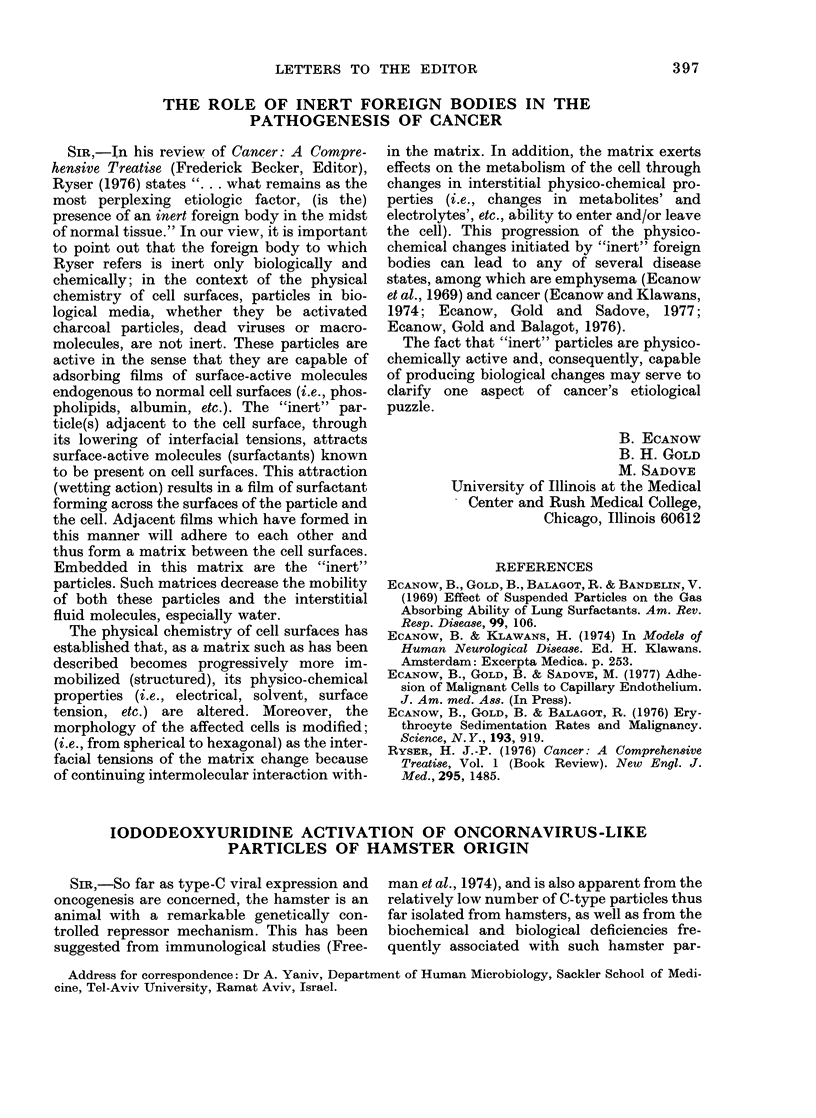# The role of inert foreign bodies in the pathogenesis of cancer.

**DOI:** 10.1038/bjc.1977.207

**Published:** 1977-09

**Authors:** B. Ecanow, B. H. Gold, M. Sadove


### THE ROLE OF INERT FOREIGN BODIES IN THE PATHOGENESIS OF CANCER

SIR,—In his review of *Cancer: A Comprehensive Treatise* (Frederick Becker, Editor), Ryser (1976) states "... what remains as the most perplexing etiologic factor, (is the) presence of an *inert* foreign body in the midst of normal tissue." In our view, it is important to point out that the foreign body to which Ryser refers is inert only biologically and chemically; in the context of the physical chemistry of cell surfaces, particles in biological media, whether they be activated charcoal particles, dead viruses or macromolecules, are not inert. These particles are active in the sense that they are capable of adsorbing films of surface-active molecules endogenous to normal cell surfaces (*i.e.*, phospholipids, albumin, *etc.*). The "inert" particle(s) adjacent to the cell surface, through its lowering of interfacial tensions, attracts surface-active molecules (surfactants) known to be present on cell surfaces. This attraction (wetting action) results in a film of surfactant forming across the surfaces of the particle and the cell. Adjacent films which have formed in this manner will adhere to each other and thus form a matrix between the cell surfaces. Embedded in this matrix are the "inert" particles. Such matrices decrease the mobility of both these particles and the interstitial fluid molecules, especially water.

The physical chemistry of cell surfaces has established that, as a matrix such as has been described becomes progressively more immobilized (structured), its physico-chemical properties (*i.e.*, electrical, solvent, surface tension, *etc.*) are altered. Moreover, the morphology of the affected cells is modified; (*i.e.*, from spherical to hexagonal) as the interfacial tensions of the matrix change because of continuing intermolecular interaction within the matrix. In addition, the matrix exerts effects on the metabolism of the cell through changes in interstitial physico-chemical properties (*i.e.*, changes in metabolites' and electrolytes', *etc.*, ability to enter and/or leave the cell). This progression of the physico-chemical changes initiated by "inert" foreign bodies can lead to any of several disease states, among which are emphysema (Ecanow *et al.*, 1969) and cancer (Ecanow and Klawans, 1974; Ecanow, Gold and Sadove, 1977; Ecanow, Gold and Balagot, 1976).

The fact that "inert" particles are physico-chemically active and, consequently, capable of producing biological changes may serve to clarify one aspect of cancer's etiological puzzle.

B. ECANOW
B. H. GOLD
M. SADOVE
University of Illinois at the Medical Center and Rush Medical College,
Chicago, Illinois 60612

REFERENCES

ECANOW, B., GOLD, B., BALAGOT, R. & BANDELIN, V. (1969) Effect of Suspended Particles on the Gas Absorbing Ability of Lung Surfactants. *Am. Rev. Resp. Disease*, **99**, 106.

ECANOW, B. & KLAWANS, H. (1974) In *Models of Human Neurological Disease*. Ed. H. Klawans. Amsterdam: Excerpta Medica. p. 253.

ECANOW, B., GOLD, B. & SADOVE, M. (1977) Adhesion of Malignant Cells to Capillary Endothelium. *J. Am. med. Ass.* (In Press).

ECANOW, B., GOLD, B. & BALAGOT, R. (1976) Erythrocyte Sedimentation Rates and Malignancy. *Science, N.Y.*, **193**, 919.

RYSER, H. J.-P. (1976) *Cancer: A Comprehensive Treatise*, Vol. 1 (Book Review). *New Engl. J. Med.*, **295**, 1485.